# Supplementary material for: IAN: An Intelligent System for Omics Data Analysis and Discovery
Source: bioRxiv. 2025 Mar 10:2025.03.06.640921. Preprint. [Version 1] doi: 10.1101/2025.03.06.640921 (PMC11952324; doi:10.1101/2025.03.06.640921)
Supplement: Supplement 1 [file NIHPP2025.03.06.640921v1-supplement-1.pdf]

bioRxiv preprint doi: https://doi.org/10.1101/2025.03.06.640921; this version posted March 11, 2025. The copyright holder for this preprint (which was not certified by peer review) is the author/funder. This article is a US Government work. It is not subject to copyright under 17 USC 105 and is also made available for use under a CC0 license.

Supplementary Data

Disclaimer: Use of Large Language Models:

Authors declare that Google Gemini was used as an aid for literature review, to generate content ideas, to correct written text, as an algorithmic tool for research study, as an evaluation tool to identify anomalies in our code and text, as a coding assistant and for documentation drafting. The authors have reviewed all content generated by Gemini.

This supplementary file includes the following content:

Supplementary Table 1: Template scoring sheet (Likert scale) for Expert Human Evaluation.
Supplementary Table 2: Ground truth categories and Groundedness score for UV and BD datasets analyzed by IAN.
Supplementary Table 3: BERT based semantic similarity scores for IAN generated reports
Supplementary Figure 1: Frequency Tables and Frequency Distribution of expert human evaluation scores for IAN's analysis reports.
Supplementary Table 4: Summary statistics on human expert evaluation of IAN's reports, using the Likert Scale of 1 to 5. 1 being worst performing, 3 average performing and 5 best performing.
Supplementary Table 5: The Wilcoxon Rank-Sum test showing no statistically significant differences in the IAN's performance across datasets and expert levels.

bioRxiv preprint doi: https://doi.org/10.1101/2025.03.06.640921; this version posted March 11, 2025. The copyright holder for this preprint (which was not certified by peer review) is the author/funder. This article is a US Government work. It is not subject to copyright under 17 USC 105 and is also made available for use under a CC0 license.

Supplementary Table 1: Template scoring sheet (Likert scale) for Expert Human Evaluation.

Score	Accuracy
1	The results and recommendations are completely inaccurate
2	The results and recommendations are slightly inaccurate
3	The results and recommendations are acceptable
4	The results and recommendations are mostly accurate
5	The results and recommendations are highly accurate
	**Relevance**
1	The results and recommendations are not relevant to this study at all
2	The results and recommendations are slightly relevant to this study
3	The results and recommendations are relevant to this study
4	The results and recommendations are mostly relevant to this study
5	The results and recommendations are highly relevant to this study
	**Clarity**
1	The results and recommendations are very difficult to understand
2	The results and recommendations are difficult to understand
3	The results and recommendations are clear
4	The results and recommendations are easy to understand
5	The results and recommendations are very easy to understand
	**Trustworthiness**
1	Doesn't make sense, so doesn't seem trustable at all
2	Doesn't make much sense, so doesn't seem very trustable
3	Makes some sense, so seems somewhat trustable
4	Makes sense, so seems trustable
5	Makes perfect sense, so seems completely trustable
	**Overall Satisfaction**
1	Completely useless, so would not recommend at all
2	Somewhat useless, would not recommend to most
3	Somewhat useful, would recommend with reservations
4	Useful, would recommend
5	Very useful, would highly recommend

bioRxiv preprint doi: https://doi.org/10.1101/2025.03.06.640921; this version posted March 11, 2025. The copyright holder for this preprint (which was not certified by peer review) is the author/funder. This article is a US Government work. It is not subject to copyright under 17 USC 105 and is also made available for use under a CC0 license.

Supplementary Table 2: Ground truth categories and Groundedness score for UV and BD datasets analyzed by IAN.

	Input Tokens	**IAN-UV-Grounded**	Total reported	Groundedness score
Network Properties Score	96	9	9	100
All Responses Genes	316	62	62	100
Final Response Genes	316	22	22	100
Integrated Network Genes	316	15	15	100
System Model Genes	316	11	11	100
WikiPathway IDs	20	16	16	100
KEGG Pathway IDs	16	8	8	100
**Average**				**100**
**Total**	**1396**			

	Input Tokens	**IAN-BD-Grounded**	IAN-Total reported	Groundedness score
Network Properties Score	453	13	13	100
All Responses Genes	1503	116	116	100
Final Response Genes	1503	21	21	100
Integrated Network Genes	1503	25	25	100
System Model Genes	1503	8	8	100
WikiPathway IDs	27	21	21	100
Reactome Pathway IDs	54	48	48	100
**Average**				**100**
**Total**	**6546**			

bioRxiv preprint doi: https://doi.org/10.1101/2025.03.06.640921; this version posted March 11, 2025. The copyright holder for this preprint (which was not certified by peer review) is the author/funder. This article is a US Government work. It is not subject to copyright under 17 USC 105 and is also made available for use under a CC0 license.

Supplementary Table 3: BERT based semantic similarity scores for IAN generated reports

	IAN-UV-Similarity
All Responses	0.7688012
Final Response	0.65062845
System Model	0.71103364
**Total/Average**	**0.71015443**

	IAN-BD-Similarity
All Responses	0.81039435
Final Response	0.7935405
System Model	0.77081585
**Total/Average**	**0.791583567**

Supplementary Figure 1: Frequency Tables and Frequency Distribution of expert human evaluation scores for IAN's analysis reports.

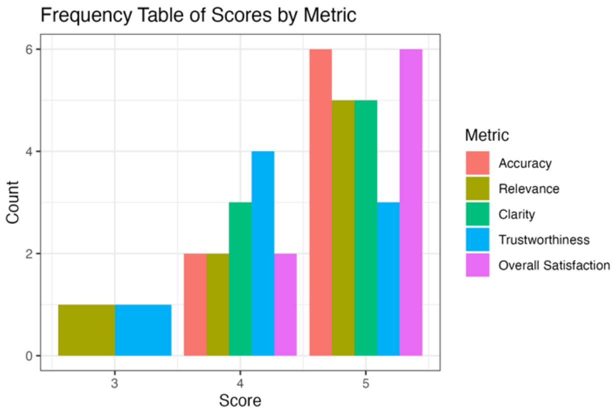

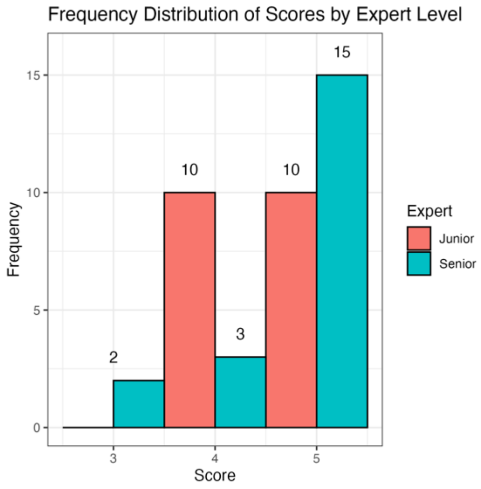

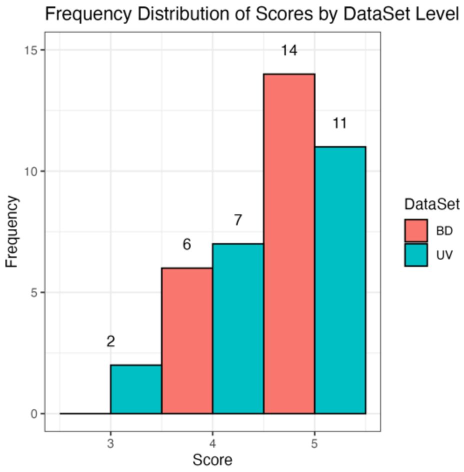

bioRxiv preprint doi: https://doi.org/10.1101/2025.03.06.640921; this version posted March 11, 2025. The copyright holder for this preprint (which was not certified by peer review) is the author/funder. This article is a US Government work. It is not subject to copyright under 17 USC 105 and is also made available for use under a CC0 license.

Supplementary Table 4: Summary statistics on human expert evaluation of IAN's reports, using the Likert Scale of 1 to 5. 1 being worst performing, 3 average performing and 5 best performing

Metric	Mean	Median	Min	Max	StdDev
**Accuracy**	4.75	5.00	4.00	5.00	0.46
**Relevance**	4.50	5.00	3.00	5.00	0.76
**Clarity**	4.63	5.00	4.00	5.00	0.52
**Trustworthiness**	4.25	4.00	3.00	5.00	0.71
**Overall Satisfaction**	4.75	5.00	4.00	5.00	0.46

Supplementary Table 5: The Wilcoxon Rank-Sum test showing no statistically significant differences in the IAN's performance across datasets and expert levels.

**Datasets: UV vs BD**		
Metric	P_value	Effect_Size
**Accuracy**	1	0
**Relevance**	1	-0.3125
**Clarity**	1	-0.25
**Trustworthiness**	1	-0.375
**Overall Satisfaction**	1	0

**Expert Level: Seniors vs Juniors**		
Metric	P_value	Effect_Size
**Accuracy**	1	0
**Relevance**	1	0.125
**Clarity**	1	0.25
**Trustworthiness**	1	0.5
**Overall Satisfaction**	1	0
